# Experience with Using the Sensewear BMS Sensor System in the Context of a Health and Wellbeing Application

**DOI:** 10.1155/2011/671040

**Published:** 2011-05-10

**Authors:** Val Jones, Richard Bults, Rene de Wijk, Ing Widya, Ricardo Batista, Hermie Hermens

**Affiliations:** ^1^Telemedicine Group, Faculty of Electrical Engineering, Mathematics and Computer Science, University of Twente, Zuidhorst building, P.O. Box 217, 7500 AE Enschede, The Netherlands; ^2^Consumer Science & Intelligent Systems, Food & Biobased Research, WUR, 6700 AA Wageningen, The Netherlands; ^3^Cluster Non-Invasive Neuromuscular Assessment (NINA), Roessingh Research and Development, P.O. Box 310, 7500 AH Enschede, The Netherlands

## Abstract

An assessment of a sensor designed for monitoring energy expenditure, activity, and sleep was conducted in the context of a research project which develops a weight management application. The overall goal of this project is to affect sustainable behavioural change with respect to diet and exercise in order to improve health and wellbeing. This paper reports results of a pretrial in which three volunteers wore the sensor for a total of 11 days. The aim was to gain experience with the sensor and determine if it would be suitable for incorporation into the ICT system developed by the project to be trialled later on a larger population. In this paper we focus mainly on activity monitoring and user experience. Data and results including visualizations and reports are presented and discussed. User experience proved positive in most respects. Exercise levels and sleep patterns correspond to user logs relating to exercise sessions and sleep patterns. Issues raised relate to accuracy, one source of possible interference, the desirability of enhancing the system with real-time data transmission, and analysis to enable real-time feedback. It is argued that automatic activity classification is needed to properly analyse and interpret physical activity data captured by accelerometry.

## 1. Introduction

It has long been recognized that the incidence of obesity and overweight in the general population is increasing to epidemic proportions, not only in the developed world, but also in the developing world, where populations have acquired “many of the unhealthy lifestyles and behaviours of the industrialized world: sedentary occupations, inadequate physical activity, unsatisfactory diets, tobacco, alcohol and drugs” [1]. Obesity and overweight are associated with increased morbidity and mortality, and many governments, HMOs, and other organizations are attempting to promote a healthier lifestyle, with weight management as a key goal. Many employers have introduced health promotion programmes for their employees, such as health checks and company-sponsored fitness schemes. 

Changing dietary and exercise patterns, however, has proved difficult to achieve. Public information campaigns about the health consequences of unhealthy eating and drinking have had little effect in changing consumer behaviour. Appealing only on the intellectual level with respect to food and drink consumption is not sufficient; unconscious emotional factors associated with eating and drinking behaviour must also be identified and addressed. In the Dutch project FOVEA [2], we are studying how to change consumer behaviour in the direction of a healthier lifestyle, with support from ICT, including ambulatory monitoring technology. As part of the project we are developing an ICT system (the FOVEA system) and a weight management application featuring a mobile monitoring and feedback system.

This paper describes an evaluation of a commercially available sensor system, the SenseWear BMS sensor [3] from BodyMedia, Inc., with respect to its suitability for ambulatory monitoring of energy expenditure in the context of this Dutch research project. The sensor system tested is designed for continuous ambulatory monitoring of energy expenditure (EE), activity, and sleep efficiency. In this paper we describe how the SenseWear device was tested by volunteers from the University of Twente acting as test users in order to gain experience with the sensor and to assess if it would be suitable for use as part of the mobile monitoring part of the FOVEA system. Three users wore the device 24/7 for a total of 11 days during normal daily life activities. The evaluation focuses mainly on user experiences of wearing the sensor continuously over a period of days and the potential use of the SenseWear system for monitoring EE. Selected data and results including visualizations and reports are presented and discussed below. User experience was positive in most respects; the only problem reported was chafing from the Velcro armband. Exercise levels and sleep patterns correspond closely to the user log relating to, for example, gym workouts and interrupted sleep patterns. Issues relating to accuracy, one possible interference factor, and the desirability of enhancing the system with real-time data transmission and analysis to enable real-time feedback to the user, are also discussed below.

Section 2 describes the FOVEA project and the requirements relating to mobile monitoring and feedback.  Section 3 presents background on the work on remote monitoring and feedback at the University of Twente. In Section 4, we describe the tested sensor system. The user testing performed in Twente is described in Section 5 with output from the SenseWear software and examples of uploaded data shown in Sections 6 and 7, respectively. Discussion of issues arising is found in Section 9, and Section 10 presents conclusions and future work. First, we describe the context of this evaluation.

## 2. The FOVEA Project

The overall objective of the FOVEA project is to investigate how best to support individuals' efforts to affect sustainable behavioural change in order to improve health and wellbeing. Specifically the project is concerned with researching, developing, and trialling a personalized workplace-based health and wellbeing application to support weight management in normal and overweight subjects. 

The requirements on the FOVEA system were determined using our scenario-based requirements elicitation methodology [4], an extension of the work of Benyon and Macaulay [5]. The full list of requirements is not reproduced here. The mobile part of the FOVEA system is designed to provide monitoring and personalized feedback relating to nutrition and activity to workers using a company canteen and will be trialled in the high-tech “Restaurant of the Future” (RoF) in Wageningen, The Netherlands. The requirements relating to monitoring of dietary and exercise behaviour of individual users include

- registration of food and drink consumption,
- estimation of energy intake,
- activity measurement, in order to derive,
- estimation of energy expenditure,
- possibility of accessing sensor data for real time and/or offline processing (either locally or remotely) all preferably in
- real time or near real time.

The SenseWear sensor was tested for suitability for the project with respect to requirements (iii)–(vi) above. Requirement (iii) is of interest here mainly for its role in the estimation of EE (requirement (iv)), Requirement (v) implies that all the raw data from the relevant sensors should be exportable. The format is immaterial, so long as it is known. Requirement (vi) is prerequisite for provision of real-time user feedback (assuming analysis and interpretation algorithms can be run in (near) real time).

With respect to requirement (iv), at the time the SenseWear BMS sensor was the only commercially available sensor we could find that provided a good estimate of daily EE compared to the gold standard of the doubly labelled water method (DLW). In one evaluation [6], the SenseWear sensor was compared with the DLW method in 45 subjects over a 10-day period. The conclusion was that the sensor “shows reasonable concordance with DLW for measuring daily EE in free-living adults. The armband may therefore be useful to estimate daily EE.” Another evaluation compared five physical activity monitors (using 21 subjects) and found that the SenseWear device gave the best estimate of total EE during walking and jogging on a treadmill at most speeds [7]. In a previous study [8], researchers at Wageningen University had good experience of the SenseWear system used in combination with a heart rate monitor to study the effects of ambient aromas on a number of physiological and behavioural factors. Results demonstrated that different ambient aromas, even at barely detectable concentrations, are associated with differential and selective effects relating to EE and food choice and even to autonomic physiological function (heart rate). 

Furthermore, as well as including analysis software capable of outputting a range of graphical views and reports, data from the sensor could be uploaded for further processing (partly addressing requirement (v) above).

The evaluation described here was conducted in an early phase of FOVEA; later phases were concerned with architecture and high-level design and (following derivation of technical requirements and detailed design) prototyping of the FOVEA system, including both mobile and fixed parts. A randomized controlled trial of the FOVEA system is planned in Wageningen, where 60 subjects with BMI of 25–30 will be randomly selected from the regular visitors to the Restaurant of the Future in Wageningen. The Restaurant of the Future provides an instrumented environment which is used in this and other projects as a testbed for interactive research in a real-life setting. The RoF infrastructure includes steerable video cameras and stereo video cameras for behavioural observation, weight scales at the checkouts, and automatic registration at point of sale terminal of individual food and drink consumptions as well as offering possibilities for altering the ambient environment in order to investigate effects of subtle changes in environmental factors on physiology and behaviour. 

The FOVEA system, currently under development, integrates components from the RoF infrastructure with the food database from the canteen supplier. In addition, specific new applications are being developed and integrated, including a tracking system for position determination and for analysis and verification of spatiotemporal behaviour patterns of users in the restaurant and a mobile application for real-time monitoring and personalized feedback to users. 

The University of Twente, with experience in developing mobile monitoring and feedback systems based on Body Area Network technology, is responsible for developing the mobile part of the FOVEA system. 

The following section gives some background on the research at Twente into remote monitoring and feedback using mobile and wireless technologies.

## 3. Body Area Networks for Healthcare: Health and Wellbeing

A multidisciplinary team of computer scientists, clinicians, and biomedical engineers at the University of Twente in the Netherlands has been researching mobile monitoring and feedback systems based on Body Area Networks (BANs) since 2001. The Twente BAN system and various healthcare applications are reported, for example, in [9–16]. Two potential health and wellbeing applications involving monitoring in extreme environments are described in [17]. 

Our definition of a health BAN is a network of communicating devices (e.g., sensors, actuators, etc.) worn on, around, or in the body which provides mobile health services to the user. For telemonitoring applications, the patient wears a BAN equipped with biosensors and possibly other devices (e.g., alarm button and positioning device) whose output is processed and transmitted to a remote (healthcare) location. The BAN data may be processed by humans, automatically, or a combination of the two, depending on the requirements of the specific application. For example, a remote healthcare professional can view a multimedia display including graphical and numerical representation of multiple biosignals and other measurements of the patient and their environment, or selectable subsets of the same kind (either in real time or stored). By including a feedback loop and actuation as well as sensing, monitoring services can be augmented with feedback and control enabling teletreatment services. Such services, especially when automated or semiautomated, require accurate and reliable processing, transmission, and interpretation of the output of multiple biosignal sources in combination with context sources which may include visual, auditory, text, and other types of information. In various BAN applications, we have combined output from multiple sensors and context sources and delivered feedback and treatment to the patient via multiple modalities including tactile, text, auditory signals, and images. 

The first prototype health BAN was implemented and trialled during the IST project MobiHealth [18]. The core BAN device, the Mobile Base Unit (MBU), acts as a communication gateway to other networks and takes care of local storage and processing. The MBU has been implemented on a number of different PDAs and smart phones. During MobiHealth, an m-health service platform and a number of variants of the health BAN, equipped with different sensor sets, were trialled in four European countries with various biosignals monitored and transmitted to remote healthcare centres over GPRS and UMTS. The nine trials in MobiHealth included telemonitoring for cardiology and COPD (respiratory insufficiency) patients, for pregnant women, for casualties in trauma care, and a professional ABN for ambulance paramedics. 

BAN development continued in the Dutch FREEBAND Awareness project [19], the European eTen project HealthService24 [20], and the European eTen project MYOTEL [21]. Awareness focussed on neurology applications (epilepsy, spasticity, and chronic pain) and addressed the issue of adding context awareness to BAN applications. In Awareness teletreatment, services were introduced alongside telemonitoring services. In the Myotel project, a prototype myofeedback-based teletreatment service which enabled patients with neck/shoulder complaints to receive personalised remotely supervised treatment during daily activities was developed. Over the course of these projects, we gained experience of signal processing and interpreting the output from different combinations of sensors and other devices. Sensors which have been integrated into the health BAN to date include electrodes for measuring ECG and EMG, pulse oxymeter, various motion sensors (step counter, 3D accelerometer), temperature, and respiration sensors. Other devices which have been incorporated into the BAN include positioning devices and a multimodal biofeedback device which measures surface EMG and gives feedback in the form of vibration and auditory signals. In this case, the biofeedback device which was incorporated into the BAN could also operate as a standalone device and was already available in this capacity as a commercial product.

With FOVEA, the work in the health and wellbeing domain was extended to providing ambulatory monitoring and personalised user feedback in the weight management application. The Fovea mobile application currently under development is designed to register food and drink selections and physical activity and provide feedback and advice tailored to the individual's specific weight management goals. The application will enable the user to choose, amongst the available lunch items in a restaurant, the ones that are in line with his dietary plan, as elaborated with a nutritionist, and his daily energy budget. The user's physical activity is monitored throughout the day, and energy expenditure is estimated in Kcal. This information is then used to calculate the energy budget, taking also into account the energy intake, which the user can spend on food items during lunch. The system also incorporates a Bluetooth beacon discovery process which enables the user to locate his office, restaurants, buffets, and weight scales within a workplace. Once a restaurant is discovered and selected, its floor plan is displayed to the user. This floor plan highlights those buffets which contain items that are compliant with the individual's preselected healthy lunch compositions. Once a given buffet is selected, all the lunch items it contains are displayed on the smart phone screen, with those which are not compliant with his healthy lunch composition highlighted, but leaving the decision ultimately to the user. When a food item is selected, its detailed information can be visualized on the smart phone, so that an informed decision can be made. If a lunch item is then confirmed, feedback is given to the user according to its impact in the available energy budget, and the lunch item information is stored in the user's food diary. Finally, the system also allows monitoring the amount of time the user takes to eat his lunch, providing feedback to ensure a properly timed meal “mindful eating.” 

In this context, we evaluated the SenseWear BMS sensor system as a possible BAN component or even as a substitute for the Twente BAN with respect to the requirements stated in Section 2.

## 4. The SenseWear BMS

**Figure 1:**
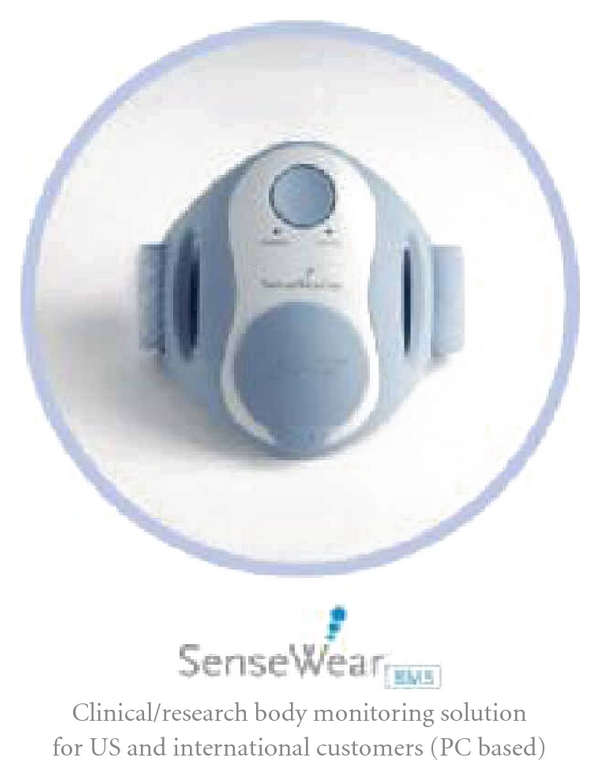
The SenseWear BMS. Source: http://www.sensewear.com/.

**Figure 2:**
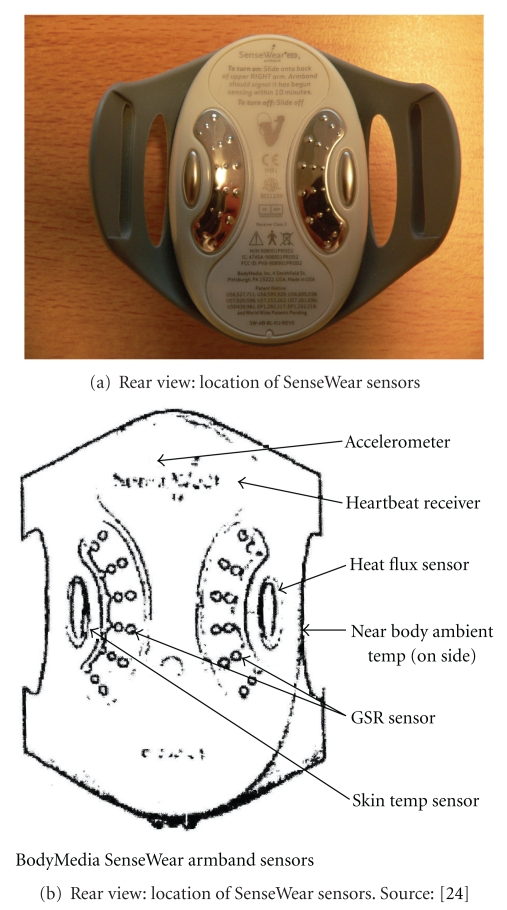


The SenseWear BMS sensor system is a commercially available sensor system from BodyMedia, Inc. designed to continuously monitor EE, activity, and sleep efficiency. It is intended for ambulatory use by patients, in consultation with their physicians, for monitoring and assessing activity levels and sleep patterns. According to the company website, the system is not intended for use as a diagnostic tool, and a typical use would be “as an assessment tool, to set a metabolic benchmark after a one-week monitoring period.” Several sensors are incorporated into a single device which is worn on the back of the upper right arm over the triceps muscle and held in place by a Velcro armband. According to [22], the sensors are 2-axis accelerometer, a heat flux sensor, a galvanic skin response sensor, a skin temperature sensor, and a near-body ambient temperature sensor.  Figure 1 shows the SenseWear device, and Figure 2 shows the rear view showing the arrangement of sensors. The variant shown in Figure 2(b) also includes a “heartbeat receiver.” The website also shows a wrist-worn feedback device (in the US model), but this was not present in the model tested.

Following configuration, the system starts up automatically when the user puts it on and only when the sensors have made a secure contact with the skin. The user can press the button to register a time stamp at any time.

According to the manufacturer's website, the most important derived parameters (calculated or estimated from data gathered by the sensors) are total energy expenditure in calories, active energy expenditure, physical activity (duration and levels measured in metabolic equivalents (METs)), and sleep duration and efficiency. According to [23], “Data from a variety of parameters including heat flux, accelerometer, galvanic skin response, skin temperature, near-body temperature, and demographic characteristics including gender, age, height, and weight are used to estimate EE using proprietary equations developed by the manufacturer.” 

 After wearing the system and registering sensor data, the data can be uploaded to a PC. Optionally all data can be wiped from the device after upload. Hence, the device can be used subsequently by a different patient (and possibly a different clinician). The device gives warning of battery failure and of memory full.

Analysis software on a dongle license key (enforcing single PC use at any one time per unit) and a USB cable are included with the system. The proprietary analysis software that accompanies the device can be run on a PC to perform various data analyses and produce on screen visualizations and reports. The reports can be saved to pdf files. There is one software application for the user (the SenseWear software), which enables the user to configure the system and to save and retrieve physiological and lifestyle data collected by the device. Another software application for use by the clinician (the SenseWear Professional software) additionally organizes the data and generates reports and visualizations in the form of graphs and permits export of data for further analysis in the form of Excel spreadsheets.

## 5. User Testing

The SenseWear device was tested by volunteers from the University of Twente acting as test users. Three users wore the device 24/7 for a total of 11 days over a period from July to September 2009. The first 3 days were a pilot, and the last 8 days were the official test, the latter yielding nearly 8∗24 = 192 hours of data for the same subject (subject 3). The device was worn during sleep and removed only when taking a shower. Subject 3 kept a log of activities during the trial.

Of greatest interest in the context of the FOVEA project is EE expressed in calories and metabolic equivalents (total and average METs) and the duration and intensity of physical activity. Activity levels can be displayed graphically as well as numerically.

Following our trial, we uploaded the data, performed various explorations using the professional software, and captured screenshots of the visualizations as described in the following sections.

## 6. Example Output

**Figure 3:**
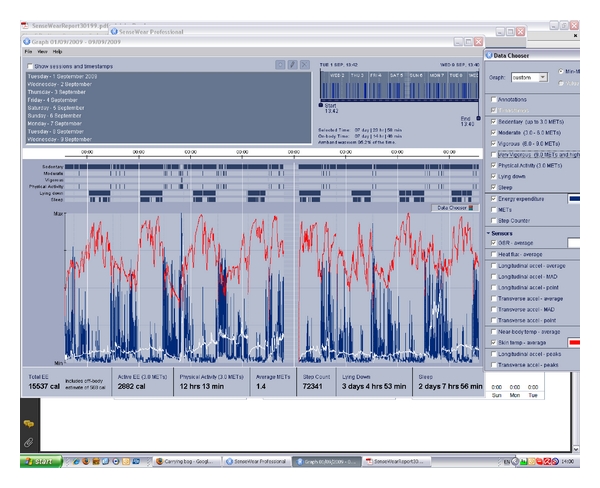
Screenshot of activity and selected sensor readings over one week.

**Figure 4:**
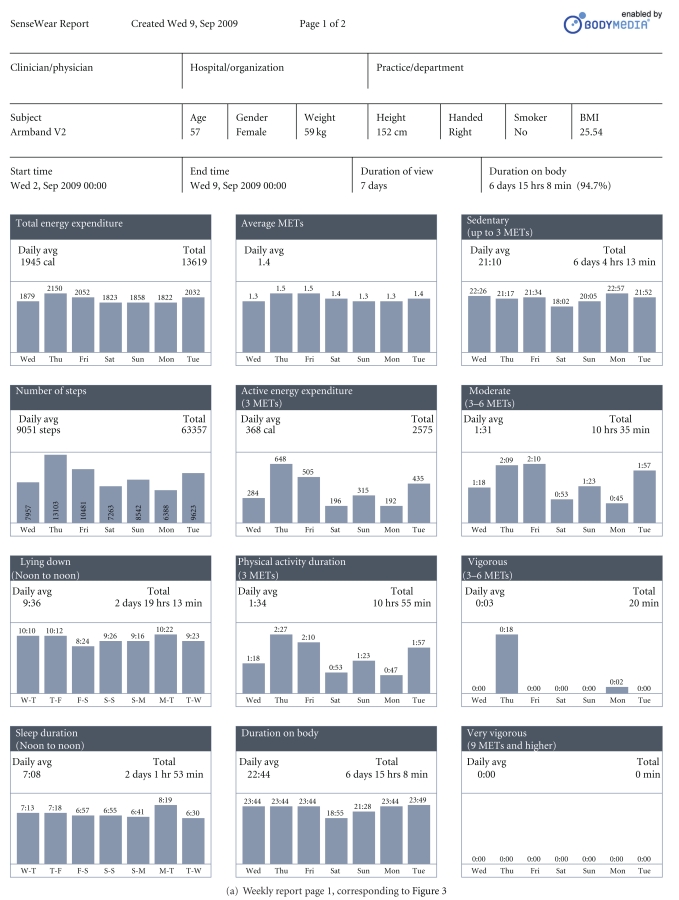

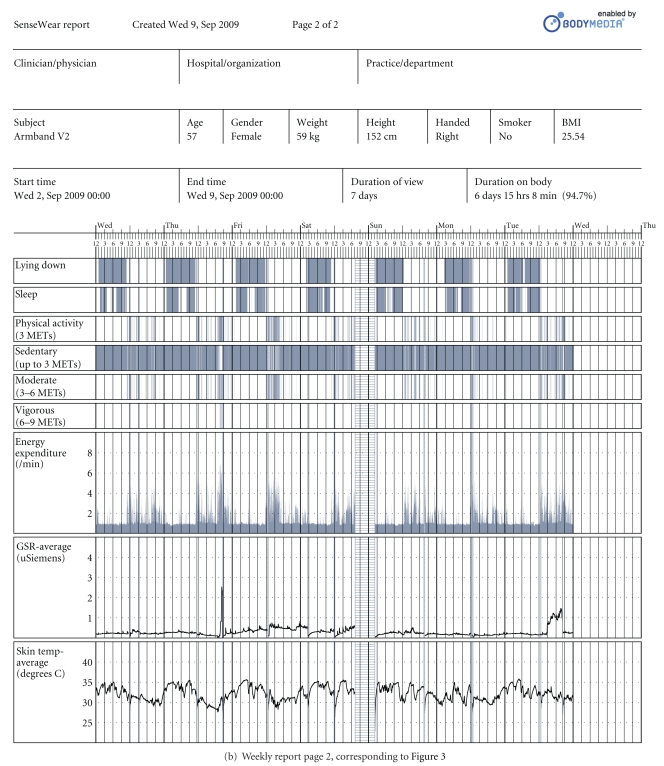


**Figure 5:**
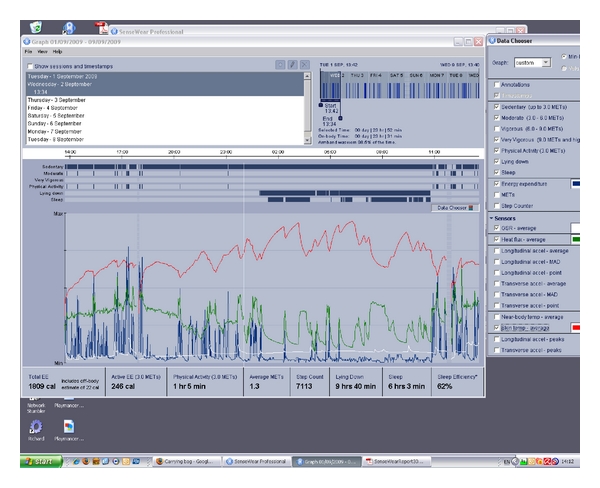
Pattern over one day (note activity levels around 17.00 at the time the subject log reports a stressful drive in rush hour traffic).

**Figure 6:**
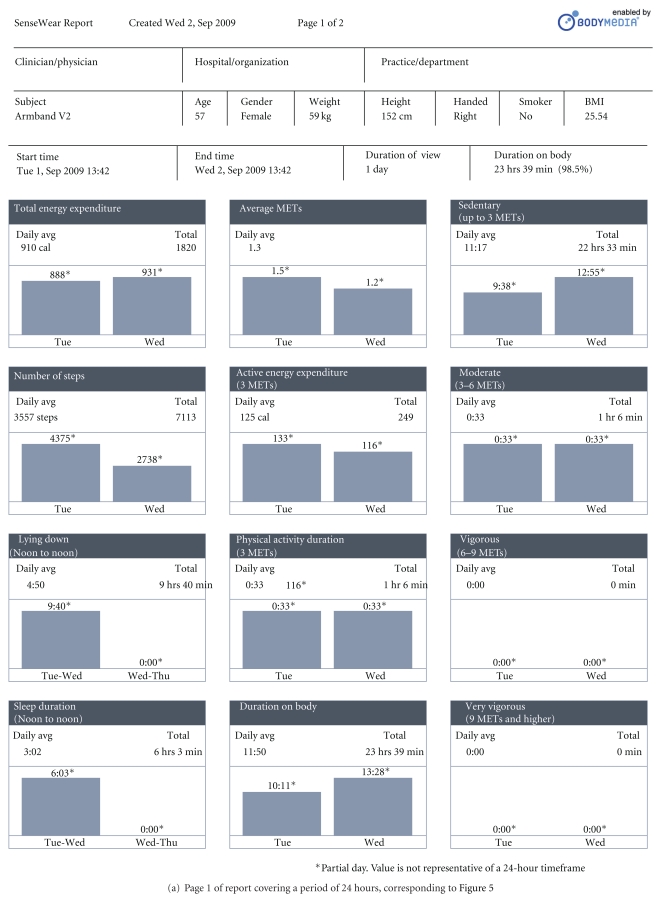

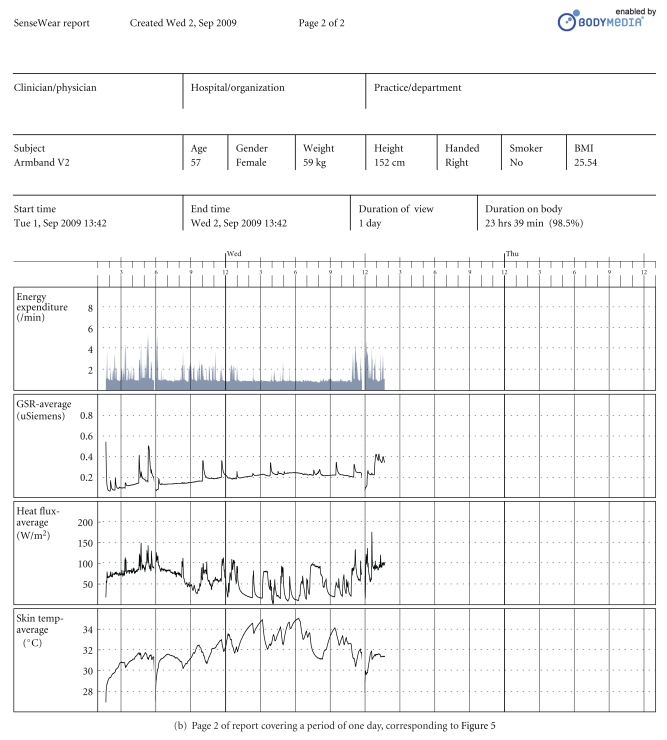


**Figure 7:**
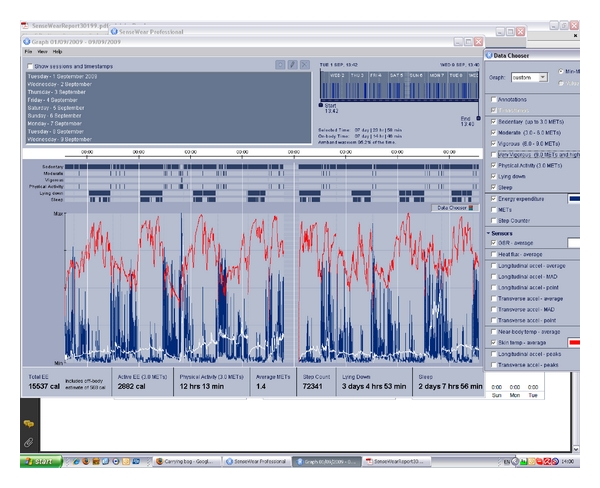
Pattern over the whole 8 days.

**Figure 8:**
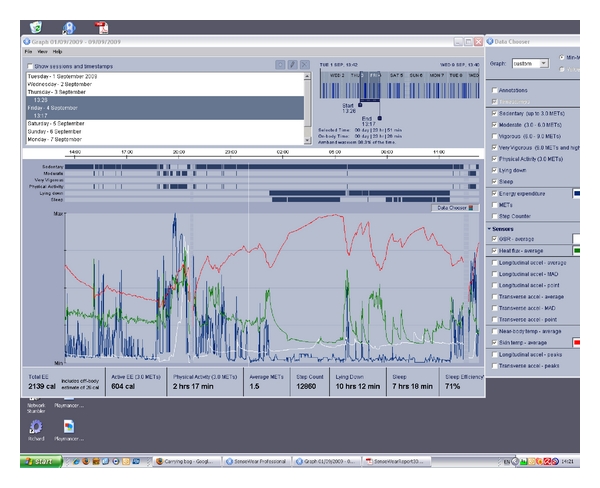
Pattern over 1 day (note the workout at the gym around 20.00).

Figures 3–8 show example output (visualizations and reports) from the SenseWear software for subject 3.  Figure 3, shows a display of selected parameters over a period of one week. Any arbitrary period and any combination of parameters can be selected for display. At the right hand side of Figure 3 all the sensors, and the parameters that can be derived from them, can be seen. In the on-screen visualizations, the selected parameters are superimposed and displayed on a timeline. 

Physical activity is measured by a 2-axis accelerometer. According to information received from a representative of the manufacturer in response to a set of queries we formulated based on our experiences [25], EE is estimated by means of “a relatively complex process involving all the sensors from the armband and many derived parameters.” The EE algorithm uses data from both axes of the 2D accelerometer; however, further details of this and other algorithms were not revealed because of the commercial sensitivity of these proprietary algorithms. 

Estimated EE is shown in calories and METS and the user can select by categories: sedentary (up to 3.0 METS), moderate (3.0–6.0 METS), vigorous (6.0–9.0 METS), and very vigorous (9.0 and higher). Total EE includes a correction for off-body time.

Figures 4(a) and 4(b), respectively, reproduce pages 1 and 2 of the pdf report corresponding to the visualization shown in Figure 3. The parameters selected on the visualization also appear separately and graphically in the pdf report. 

The nine graphs in Figure 4(b) correspond to the selection of nine parameters made on that occasion (see Figure 3).

Figure 5 shows the pattern over a single day (the first 24 hours of use for subject 3). 

Note activity levels around 17.00 at the time the subject log reports a stressful drive to an important appointment through rush hour traffic made worse by unexpected roadworks and diversions. 

Subject 3 is a chronic insomniac. Sleep patterns shown in Figure 5 correspond closely to the periods of sleep and wakefulness reported in the subject log. The lower right hand part of the screen shows time lying down (in bed) as 9 hours 40 minutes whilst sleep time was only 6 hours 3 minutes, yielding a sleep efficiency measure of 62%.

On the same figure, we see an increase in activity around 11 am the following morning. The user log records a short low-intensity exercise session around 11.00 am. Curiously, the stressful drive to the garage the previous evening registered a higher maximum activity level (energy expenditure): EEmax = 5.13936376571655 (with rather bursty patterns) than this exercise session with EEmax = 4.06614971160888. We return to the driving episode in the discussion section below.

Figures 6(a) and 6(b), respectively, reproduce pages 1 and 2 of the report covering a period of one day and correspond to Figure 5. 

The first page of the report (see Figure 6(a)) gives summary information on clinician and hospital, patient data, timing and duration of usage on body, and data on EE and sleep over the selected period (the first 24 hours of use). Because the user test for subject 3 started at 13.42 on the first day, the 24-hour period starts from that time. Over the 24-hour period (split over two partial days), total EE was 1820 calories. 22 hours 33 minutes was recorded as sedentary and 1 hour 6 minutes as physical activity (all at moderate exercise level, defined as 3.0–6.0 METS), accounting for the 23 hours 39 minutes duration on body during the 24-hour period. Total step count over the 24 hours was 7113. 

The second page of the report (reproduced in Figure 6(b)) gives more detailed graphical representations of the selected parameters (cf Figure 5). 

Figure 6(b) also shows that spikes in EE are sometimes echoed in the GSR trace, which would be expected due to increased transpiration. Average skin temperature rises during the afternoon and evening and peaks during the night. Skin temperature and heat flux sometimes show an apparently inverse relationship in the detail between 12 midnight and 12 noon. 

Figure 7 shows the pattern over the whole test period of eight days for subject 3, allowing diurnal patterns to be compared visually over a period of days. The armband was worn 95.2% of the time over the total period of eight days (as mentioned previously, it needs to be removed for bathing), indicating that long-term use was not a problem in this case. Disrupted sleep patterns, which correspond closely to the user log, can be clearly seen by comparison of the graphic displays for “lying down” and “sleep,” and the extent of sleep disruption can be seen by the total time lying down: 3 days 4 hour 53 minutes compared to total time sleep: 2 days 7 hours 56 minutes. One of the questions put to the manufacturer's representative was “How is lying down distinguished from sleep?” The response was that the algorithm uses all the sensors and many derived parameters, but further information could be revealed [25]. The red trace shows average skin temperature; the white trace shows average galvanic skin response; the blue trace shows energy expenditure.

Figure 8 shows the pattern over another single day. The red trace shows average skin temperature; the white trace shows galvanic skin response average; the blue trace shows energy expenditure; the green trace shows heat flux (average). The activity pattern around 20.00 corresponds to a workout at the gym as reported in the user log and corresponds to a rise (several peaks) in EE and a steady rise in average skin temperature both followed with a short time lag by rises in heat flux and then GSR. 

## 7. Uploaded Data

Data from the SenseWear device can be uploaded to a PC in the form of an Excel spreadsheet. Four kinds of data are included

- timestamped preprocessed data derived from analysis of sensor data,
- subject information,
- summary information,
- clinician information.

### 7.1. Timestamped Preprocessed Data

Table 1 shows an extract of the timestamped pre-processed sensor data for subject 3. The data is recorded once per minute. The table is split into two in order to fit on the page. 

### 7.2. Subject Information

The following information can be entered when the user configures the device:

- subject,
- age,
- height,
- weight,
- gender,
- handedness,
- smoker,
- serial number,
- BMI.

### 7.3. Summary Information

Table 2 shows the entire summary sheet for all the data for subject 3 (8 days). The table is split into two in order to fit on the page. 

The first day was a short day with the experiment starting on day 1 at 13.42, and hence the sensor was worn only for just over 10 hours up until midnight. Similarly, the last day was also a short day as the experiment ended at 13.39 on day 9. It can be seen that the subject was able to wear the device up to 98.9% of the time. Hours off body on the 5th of September corresponds to the entry on the log where the subject removed the device when attending a party so as not to attract attention. The software compensates for time off body by estimating EE during that time.

### 7.4. Clinician Information

The following information can be entered at the start of use:

- clinician,
- organization,
- department,
- notes.

Obviously, the four kinds of data from the spreadsheets can easily be input to other applications, however, only retrospectively in the case of the system tested. Moreover, the raw data is not available. These two facts limit the potential utility of the device if the ambition is to perform real-time analysis and provide real time feedback to users, as is the intention in the FOVEA project.

## 8. User Observations on the Use of the SenseWear System

User impressions were that the device is very comfortable and unobtrusive and can be easily worn 24/7. The simple and intuitive interface, using a single button, vibratory and auditory feedback (two different patterns of beeps), and two lights, was considered to be easy to use and well designed by the standard expected for quality consumer electronics products. Memory full is indicated by a red light appearing above “memory,” and battery low is indicated by a red light appearing above “battery” on the armband. Subject 3 reported that wearing the device did not interfere with sleep or other normal daily life activities, except that the device has to be taken off for showering/bathing. However, after two days' continuous wear (apart from in the shower), subject 3 found that friction from the edge of the Velcro armband caused skin damage serious enough to stop the experiment. In the event, the user found a makeshift solution by cutting a strip from a compression bandage and inserting it between skin and Velcro armband, carefully avoiding interfering with the sensor-skin contact. The result was effective (if not aesthetically pleasing!), and the experiment continued as planned.

Exercise levels and sleep patterns (e.g., as shown in Figure 3) corresponded very closely to the manual log kept by the subject, relating to, for example, gym workouts, interrupted sleep patterns, and the stressful drive to reach an appointment though rush hour traffic with unexpected diversions.

## 9. Discussion

User experience as reported was positive with the exception of the skin problem caused by chafing of the Velcro armband. As described above, a work around was easily found, so that the experiment could continue.

The proprietary algorithms used by the SenseWear software are not in the public domain; hence, it is difficult to tell exactly how energy expenditure and other derived parameters are calculated or estimated. Published evaluation studies mainly apply an experimental approach to compare the results delivered by the SenseWear system with recognized standards, namely the doubly labelled water method (DLW) and indirect calorimetry (IC), and/or with other products, under controlled conditions. 

A number of evaluation studies have been conducted on different models in the products range. One issue raised is the point that the accuracy of energy expenditure estimations appears to be affected by many different parameters. One study examined the validity of the SenseWear Pro 2 armband to assess energy expenditure during various modes of physical activity in twenty-four healthy female and male adolescents. Energy expenditure measured by the armband during treadmill at different speeds and gradients and cycle ergometer exercise was compared with respiratory metabolic system (RMS). 

The main findings were that the SenseWear Pro 2 “significantly underestimated energy expenditure during cycle ergometer exercise at the low (1.53 + 0.60 kcal·min^−1^; *P* < .001) and moderate (2.48 + 0.95 kcal·min^−1^; *P* < .001) intensities and for total energy expenditure (19.11 + 7.43 kcal; *P* < .001) in both the female and male subjects….” (However, this would be expected if the armband was worn on the arm during cycling tests) and in the treadmill exercise “there were no significant differences between measures of energy expenditure during treadmill walking at 3.0 mph, 0% incline in female and male subjects.” However, the (SenseWear Pro 2) significantly underestimated measures of energy expenditure at 4.0 mph, 0% grade (0.86 + 0.84 kcal·min^−1^; *P* < .001); 4.0 mph, 5% grade (2.13 + 1.40 kcal·min^−1^; *P* < .001); 4.5 mph, 5% grade (2.97 + 1.56 kcal·min^−1^; *P* < .001) and for total energy expenditure (23.66 + 14.92 kcal; *P* < .001) during treadmill exercise in female and male subjects [26].

Other studies, for example, [27, 28] arrived at favourable conclusions. In [28], the subjects were children aged seven to ten years, and it is pointed out, however, that exercise-mode-specific algorithms may be necessary for adults as well as children in order to achieve more accurate measures of energy expenditure and that accuracy of the algorithms in other ethnic populations is unknown (their study population was “primarily Caucasian and representative of rural Pennsylvania”). SenseWear subsequently developed age-specific algorithms.

A comparison of three products was made in 2009 [29], concluding that all three needed further development but that the SenseWear device might be more feasible for use under free-living conditions, although it was less accurate than another product (the IDEEA device) in assessing energy cost. 

In an earlier study in 2004 [30], the SenseWear armband's EE estimates were compared with IC (indirect calorimetry) in adults during rest and exercise. The conclusion was that “The SWA (*SenseWear Armband*) provided valid and reliable estimates of EE at rest and generated similar mean estimates of EE as IC on the ergometer; however, individual error was large. The SWA overestimated the EE of flat walking and underestimated inclined walking EE.” 

In 2006, a comparison of the energy expenditure estimates of the SenseWear Pro 2 against the IC method on adults during rest and during three exercise sessions was conducted [23]. In this study, the experimental group consisted of obese adults; the controls were lean and overweight adults. The REE (resting energy expenditure) estimated by the SenseWear device showed high correlation and very good agreement with measured IC in lean and overweight adults but showed poor accuracy in obese adults, especially those with high REE, in rest and in exercise. This is not so serious for the FOVEA project since overweight subjects will be included, but clinically obese subjects will be excluded.

According to the *Instructions For Use* [31], the device should be worn on the back of the upper right arm. We presume that lower activity levels are expected in the nondominant arm. There is an option to record handedness in the subject information although this is not enforced. It would seem then that activity levels will be underestimated for left-handed users, and interindividual comparisons would need to correct somehow for handedness. 

We cannot see any way to interface the model tested to a PDA or smartphone; hence, it appears that it would be difficult or impossible to incorporate it into a body area network. The data is uploaded to a PC via USB cable, using the proprietary SenseWear software; hence, the use of this model in the FOVEA project, for example, would involve importing the SenseWear output files retrospectively and periodically from a PC to an FOVEA application (e.g., the application running on a mobile device giving personalised feedback to the user). So although the model tested cannot communicate with a PDA/smartphone, it could be used standalone if data is merged offline and retrospectively. It could be used in this way, for example, to show a subject's recent history of energy expenditure and compare that with current energy intake if the subject uploads data regularly, for example, once per day. 

It is possible to incorporate additional sensors wirelessly in one model: “The functionality of the SenseWear Armband can be expanded with the SenseWear Transceiver. This tiny programmable module can be integrated into digital products such as a pulse oximeter to enable 2-way communication with the SenseWear Armband” [22]. The model tested by us, however, has no real-time data transmission for upload of data, so the user must perform periodic uploads to a PC via a USB connection. 

Another drawback in the model tested is that although the data can be uploaded to a PC, the raw data is not accessible and only pre-processed data is uploaded (see Table 1). This severely limits the kinds of postprocessing which can be performed. 

We return to the incident when driving a car apparently involved higher activity levels than a low-intensity exercise session. It is true that driving a car, especially one with a manual gearbox, does involve physical work, and the level of activity resulting will vary with driving style. In any case, however, the accelerometer will be affected by acceleration forces, deceleration forces, centrifugal forces, and so on caused by the motion of the car, thereby artificially inflating the readings. Without the user log in this case, this episode could have gone unnoticed. Whether and how the algorithms recognize specific activities and correct for them is difficult to tell without access to the algorithms, but the comparison of the driving episode and the exercise session raised our suspicions. Certainly it would be possible in principle to recognize an *in moving vehicle* setting (on the basis of vibration, e.g.) and possibly to correct for this effect by making some estimate based on certain assumptions. Alternatively the effect could be factored out by subtraction by using a fixed sensor placed in the same vehicle. The latter is only practicable in experimental situations, not in free-living conditions, but could yield interesting experimental results by quantifying the effect under different driving conditions. The question was posed to the manufacturer “Can your algorithms for EE detect when the subject is in a moving vehicle? If so, how? And do your algorithms compensate for the acceleration forces, and so forth, due to vehicular motion?? If so, how?” The response was “yes,” but details of the algorithms could not be given for the same reasons as previously stated [25].

This moving vehicle scenario is one example which underlines the desirability in some circumstances of augmenting sensor data with contextual information in order to arrive at proper interpretation of (multiple streams of) sensor data. Manual logs by users are very impractical for use in everyday life, and even augmenting sensor data with video brings huge additional time costs to processing and analysis. The need for automated activity recognition and classification seems self-evident. This challenging research area is beginning to yield results by applying techniques such as Hidden Markov Modeling and machine learning to the classification of activity data registered during long episodes of human motion. In [32, 33], for example, the activities examined involved different ways of lifting two different loads (from the left, from the right, lifting with straight legs and with bent knees), putting the load down, walking, standing, and sitting [32]. These activities are at a lower level of abstraction than those we are addressing in FOVEA. However, the techniques applied are worth investigating and could be combined with some higher-level abstraction mechanism applied to motion and other data to infer higher-level activities.

## 10. Conclusions and Future Work

In summary, the user experience of the SenseWear sensor was very positive. Sleep and activity data seemed to correspond well with the user logs although the driving episode suggests further investigation of methods for detection of, and correction for, contextual parameters such as being in a moving vehicle. 

The SenseWear sensor was tested for suitability for the FOVEA project with respect to requirements (iii)–(vi) as detailed in Section 2 above, namely,

(iii) activity measurement,
(iv) estimation of energy expenditure,
(v) possibility of accessing sensor data for real time and/or offline processing (either locally or remotely) all preferably in
(vi) real time or near real time.

Requirements (iii) and (iv) were met (with some reservations concerning accuracy in specific contexts) by the model tested. Regarding requirements (v) and (vi), we cannot see any way to interface the model tested to a PDA or smartphone; hence, it appears that it would be difficult or impossible to incorporate it into a body area network. Raw data is not accessible, only pre-processed data can be uploaded which severely limits the kinds of postprocessing which can be performed. Without raw data and access to the algorithms, we are in something of a black box situation. For an external application such as ours with a need to take output from the sensor and to give information and feedback to the user in real time, this model does not meet our requirements for this project. 

Since the user tests reported here were conducted, however, the manufacturer has introduced a model with a Bluetooth interface [34]. In this model, two factors which are prerequisites for giving user feedback in real time or near real time are now implemented: firstly, the algorithms now operate in real time (some algorithms need to be applied retrospectively to cumulative data, precluding real-time feedback); secondly, the sensor can communicate in real time with its display device and update it with respect to the parameters concerned with physical activity, step count, and EE [35]. This new model therefore would appear to go some way towards addressing requirements (v) and (vi), but this would need to be investigated in more detail. However, this new model does not transmit all of the raw data, although there was a hint that future models might.

Irrespective of whether this range of sensors is used in the FOVEA project, it still seems to us to have interesting and useful capabilities, not only for estimation of EE, but also for activity measurement and sleep analysis, and is worthy of further study. Looking at the results, it is evident that the sensor can produce useful data, although with what accuracy is difficult to determine without access to the raw sensor data and algorithms. As mentioned above, literature does report favourable findings for estimation of energy expenditure in comparison to indirect calorimetry and the doubly labelled water method, but the questions over accuracy in different contexts (such as different exercise modes and the moving vehicle setting) highlight the need to supplement sensor data with information from a variety of context sources. In general, which context sources are needed depends on many factors including the precise application, the capabilities of the sensor or sensors used, and the sophistication of the interpretation algorithms. 

In future work, we plan to conduct further experiments within and beyond FOVEA using the new model of the SenseWear system, and other sensors including an activity sensor being developed by Roessingh Research and Development (RRD) [32, 33] which combines 3D acceleration data with 3D angular velocity data (the combination gives better performance in activity recognition tasks than accelerometry alone [32]) and for which raw data and algorithms are accessible. We also plan to experiment with onboard accelerometers in new generation mobile phone platforms.

## Figures and Tables

**Table 1a:** (a) Extract of the timestamped pre-processed sensor data for subject 3 (columns 1–8).

Time (GMT+02:00)	Lying down	Physical activity	Moderate	Sedentary	Very Vigorous	Vigorous	Sleep
2009-09-01 13:42:00,000	0	0	0	1	0	0	0
2009-09-01 13:43:00,000	0	0	0	1	0	0	0
2009-09-01 13:44:00,000	0	0	0	1	0	0	0
2009-09-01 13:45:00,000	0	0	0	1	0	0	0
2009-09-01 13:46:00,000	0	0	0	1	0	0	0
2009-09-01 13:47:00,000	0	0	0	1	0	0	0
2009-09-01 13:48:00,000	0	1	1	0	0	0	0
2009-09-01 13:49:00,000	0	1	1	0	0	0	0
2009-09-01 13:50:00,000	0	0	0	1	0	0	0
2009-09-01 13:51:00,000	0	0	0	1	0	0	0
2009-09-01 13:52:00,000	0	0	0	1	0	0	0
2009-09-01 13:53:00,000	0	0	0	1	0	0	0
2009-09-01 13:54:00,000	0	0	0	1	0	0	0

**Table 1b:** (b) Extract of the timestamped pre-processed sensor data for subject 3 (columns 9–14).

Energy expenditure	GSR average	Heat flux average	Skin temp average	Annotations	Timestamps
1,140285611	0,536105335	18,57492638	26,9446907		
1,039066195	0,401054025	30,32817268	27,81936646		
2,510075331	0,302013814	54,6265564	28,19648743		
1,171944857	0,223543122	57,00035858	28,32251167		
1,135450482	0,170754269	53,58623123	28,51185989		
1,162263155	0,137766883	54,8740387	28,6593895		
3,854850769	0,117243603	79,31423187	28,72268677		
2,998823404	0,104050949	75,92498779	28,70158386		
1,168393016	0,092324734	65,98029327	28,80715179		
1,064719796	0,083530433	64,71324921	28,91283989		
1,167660356	0,079866238	68,97907257	28,9551506		
1,139359117	0,074736446	67,09690094	29,01865005		
1,084661007	0,07107237	66,3093338	29,06101036		

**Table 2a:** (a)

Date	Hours of armband data	Hours offbody	Percent onbody	Total EE	Measured EE	Offbody EE	Measured active EE
2009-09-01	10:11	0:07	98,9%	888	881	7	133
2009-09-02	23:44	0:16	98,9%	1879	1863	16	284
2009-09-03	23:44	0:16	98,9%	2150	2134	16	648
2009-09-04	23:44	0:16	98,9%	2052	2036	16	505
2009-09-05	18:55	5:05	78,8%	1823	1509	314	196
2009-09-06	21:28	2:32	89,4%	1858	1702	156	315
2009-09-07	23:44	0:16	98,9%	1822	1806	16	192
2009-09-08	23:49	0:11	99,2%	2032	2021	11	435
2009-09-09	13:27	0:13	98,4%	1030	1017	13	174

Totals	182:46	9:12	95,2%	15537	14969	568	2882

**Table 2b:** (b)

Physical activity threshold	3,0	
Sedentary	0,0	3,0
Moderate	3,0	6,0
Vigorous	6,0	9,0
Very vigorous	9,0	and up

**Table 2c:** (c)

Physical activity	Steps	Lying down	Measured sleep	Average METs	Sedentary	Moderate	Vigorous	Very vigorous
duration								
0:33	4375	0:00	0:00	1,47	9:38	0:33	0:00	0:00
1:18	7957	9:40	6:03	1,33	22:26	1:18	0:00	0:00
2:27	13103	10:10	7:13	1,52	21:17	2:09	0:18	0:00
2:10	10481	10:12	7:18	1,45	21:34	2:10	0:00	0:00
0:53	7263	8:24	6:57	1,35	18:02	0:53	0:00	0:00
1:23	8542	9:50	6:57	1,34	20:05	1:23	0:00	0:00
0:47	6388	8:52	6:39	1,29	22:57	0:45	0:02	0:00
1:57	9623	10:42	8:39	1,44	21:52	1:57	0:00	0:00
0:45	4609	9:03	6:10	1,28	12:42	0:45	0:00	0:00
12:13	72341	76:53	55:56	1,39	170:33	11:53	0:20	0:00
